# Retraction: Long non-coding RNA HOTAIR acts as a competing endogenous RNA to promote malignant melanoma progression by sponging miR-152-3p

**DOI:** 10.18632/oncotarget.28822

**Published:** 2025-12-31

**Authors:** Wenkang Luan, Rubo Li, Liang Liu, Xin Ni, Yan Shi, Yun Xia, Jinlong Wang, Feng Lu, Bin Xu

**Affiliations:** ^1^Department of Plastic Surgery, Affiliated People’s Hospital of Jiangsu University, Zhenjiang, China; ^2^Department of Neurosurgery, Affiliated People’s Hospital of Jiangsu University, Zhenjiang, China; ^3^Department of Neurosurgery, Nanjing First Hospital, Nanjing Medical University, Nanjing, China; ^4^Department of Gastroenterology, Affiliated Hospital of Jiangsu University, Zhenjiang, China; ^*^These authors have contributed equally to this work

**This article has been retracted:** Oncotarget has concluded its investigation of this paper. Multiple image misrepresentations/manipulations were discovered. Specifically, the following issues were found:

- Figure 2J: The Western blot image for N-cadherin in A375 cells was horizontally and vertically flipped and reused as E-cadherin.
- Figure 2F: The image for the wound healing assay (NC 24 h) is a duplicate of a NC 24 h image in Figure 6F.
- Figure 5L: The Western blot images lack background and contain several splices. All paired total protein and phospho-proteins blots do not match.
- Figure 5G: The miR152-3p image of the transwell assay was reused in Figure 4D of article [1] and Figure 5B of article [2], both published earlier by the same first author.
- Figure 2H: The NC and si-HOTAIR panels overlap with Figure 5B of article [2].
- Figure 6H: The si-HOTAIR image of the transwell assay was reused in Figure 4D of article [1].
- Figure 6H: All transwell assay images were reused in Figure 5C of article [3], where Luan W. is a co-author.

The authors acknowledged mistakes in Figures 2H, 5G, and 6G–6I . They also admitted to the misuse of representative pictures of immunohistochemical staining when assembling Figure 7E and provided corrected figures. However, their explanation was not satisfactory, and they failed to provide the original, uncropped, and unmodified images necessary to verify the corrections. Given the extent of necessary corrections, the editorial decision was to retract the article. The authors have been notified of this decision.

Original article: Oncotarget. 2017; 8:85401–85414. 85401-85414. https://doi.org/10.18632/oncotarget.19910
